# Potential Effect of Box‐Shaped Pulmonary Vein Isolation on Persistent Atrial Fibrillation in a Patient With Severe Pectus Excavatum

**DOI:** 10.1002/ccr3.70882

**Published:** 2025-09-16

**Authors:** Takeshi Mori, Kazuo Kato, Hiroko Goto, Shinjiro Miyata

**Affiliations:** ^1^ Department of Cardiology Nagoya Tokushukai General Hospital Kasugai Aichi Japan

**Keywords:** atrial fibrillation, box‐shaped pulmonary vein isolation, cavotricuspid isthmus ablation, left atrial posterior wall, pectus excavatum, radiofrequency ablation

## Abstract

In atrial fibrillation patients with pectus excavatum, box‐shaped pulmonary vein isolation may be preferable due to left atrial posterior wall involvement. However, it needs extra roof and bottom ablation. Preoperative imaging and use of contact force‐sensing catheters with three‐dimensional mapping are vital for safe and effective procedures.

## Graphical abstract

Severe pectus exacatum (A) Caudal veiw (B) Right anterior oblique view.

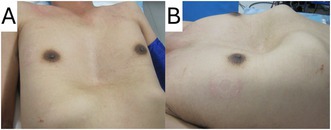

## 1 Introduction

Pulmonary vein isolation (PVI) is an established and effective treatment for atrial fibrillation (AF), regardless of whether it is paroxysmal or persistent [1, 2]. While pulmonary veins are the primary foci of AF, non‐pulmonary vein (non‐PV) foci, including the left atrial posterior wall (LAPW), have also been identified as major triggers [3]. The box‐shaped PVI (box‐PVI) technique, which isolates both the pulmonary veins and LAPW in a single isolation procedure, was first described by Kumagai and represents an advanced approach for managing AF of complex origins [4].

Pectus excavatum (PE) is the most common congenital thoracic deformity [5]. In patients with PE, the left atrium, including the LAPW, is often considerably compressed between the sternum and vertebral bodies, which may be responsible for the electrical instability resulting in AF foci. Patients with PE are at a higher risk of developing AF [6]. Despite the established relationship between PE and AF, reports on PVI in this population remain limited [7, 8, 9]. To the best of our knowledge, this is the first report of box‐PVI performed on a patient with PE, highlighting its feasibility and potential clinical value.

## 2 Case Presentation

**FIGURE 1:**
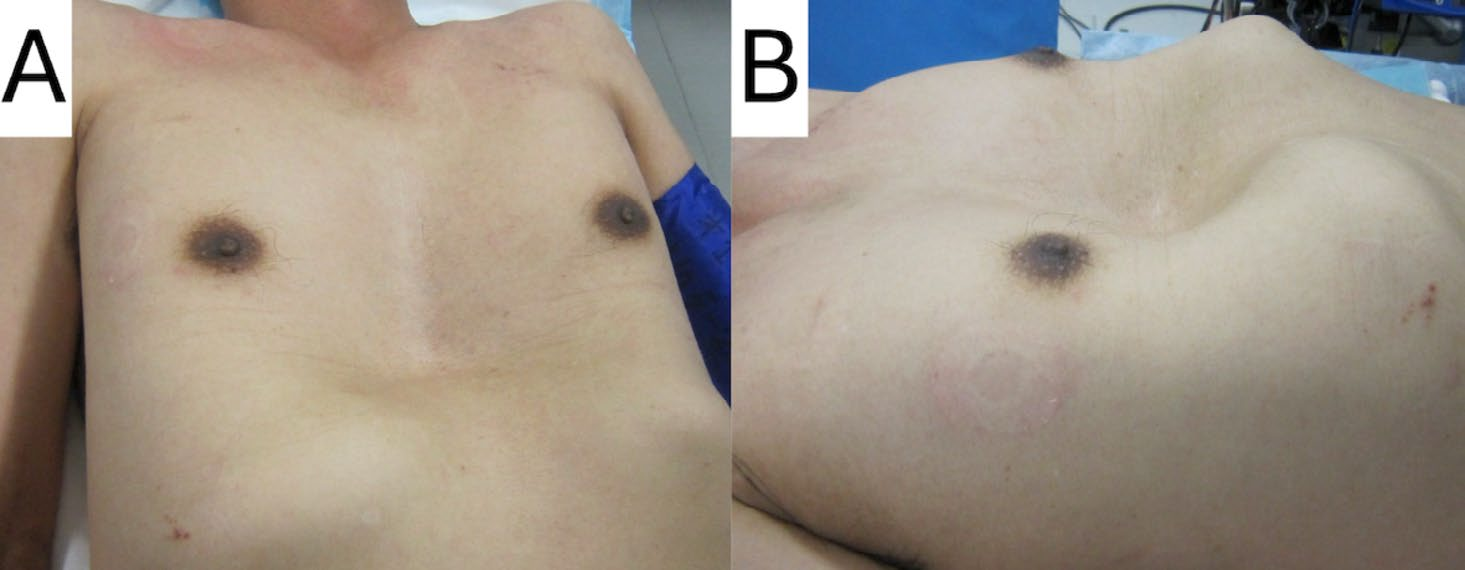
Pectus excavatum. The chest was severely sunken, exhibiting signs of pectus excavatum. (A) Caudal view. (B) Right anterior oblique.

A 57‐year‐old man with well‐controlled hypertension, diagnosed 5 years ago and managed non‐pharmacologically at a local clinic, presented at our institution with heart failure. Physical examination revealed PE (Figure 1). The patient required hospitalization at a referral institution 4 months prior to presentation. At the time of admission, AF with tachycardia was documented. Laboratory investigations revealed a B‐type natriuretic peptide level exceeding 700 pg/mL, and transthoracic echocardiography showed a mildly reduced left ventricular ejection fraction (LVEF) of 43%, accompanied by pleural effusion. Rate control with a β‐blocker, along with appropriate heart failure management, temporarily restored the sinus rhythm; however, persistent AF recurred.

**FIGURE 2:**
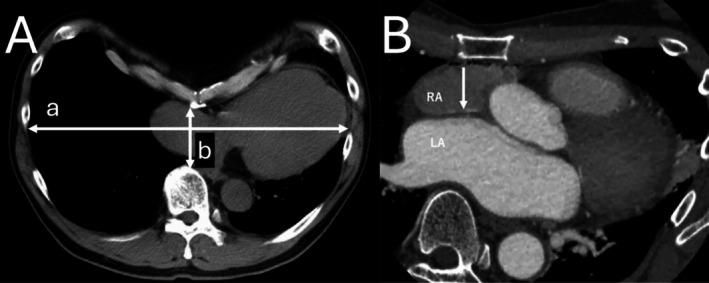
Haller index and Brockenbrough site. (A) Chest computed tomography (CT) suggests severe pectus excavatum. (a) Maximum transverse diameter: 272.84 mm. (b) Minimum anterior–posterior diameter: 51.29 mm. Haller index = 272.84/51.29 = 5.31. Cardiac CT shows a depressed thoracic cage, resulting in significant compression of the left atrium against the vertebral body. Furthermore, the spatial relationship between the inferior vena cava and heart is markedly altered, deviating from the normal anatomical configuration. (B) The white arrow indicates the direction in which the radiofrequency needle was oriented during the Brockenbrough procedure, demonstrating the necessity of advancing the needle toward the vertebral body.

Coronary angiography (CAG) revealed no abnormal anatomy or significant coronary artery stenosis. Prior to performing catheter ablation, contrast‐enhanced chest computed tomography (CT) was performed to evaluate the anatomical structure of the heart, including a pre‐procedural assessment of the left atrial morphology. Chest CT revealed marked cardiac compression between the sternum and vertebral column, with a Haller index of 5.37, consistent with severe PE (Figure 2A). As AF tachycardia is a major contributing factor to heart failure exacerbation and LVEF reduction, catheter ablation was considered the most appropriate therapeutic approach. Evidence suggests that the absence of surgical repair for PE may result in symptoms, such as dyspnea or palpitations in older adult patients [10]. Consequently, surgical treatment was recommended; however, the patient opted not to proceed with the procedure. As the previous institution did not have an affiliated cardiovascular surgery department capable of performing emergency interventions, and considering the anatomical deviations associated with PE that could increase the risk of complications during ablation, the patient was referred to our hospital, which is fully equipped to provide immediate surgical management, if needed.

## 3 Methods

All ablation procedures were performed with the patients under deep sedation using dexmedetomidine, propofol, and buprenorphine, with esophageal temperature and direct blood pressure monitoring.

### 3.1 Box‐Shaped Pulmonary Vein Isolation

**FIGURE 3:**
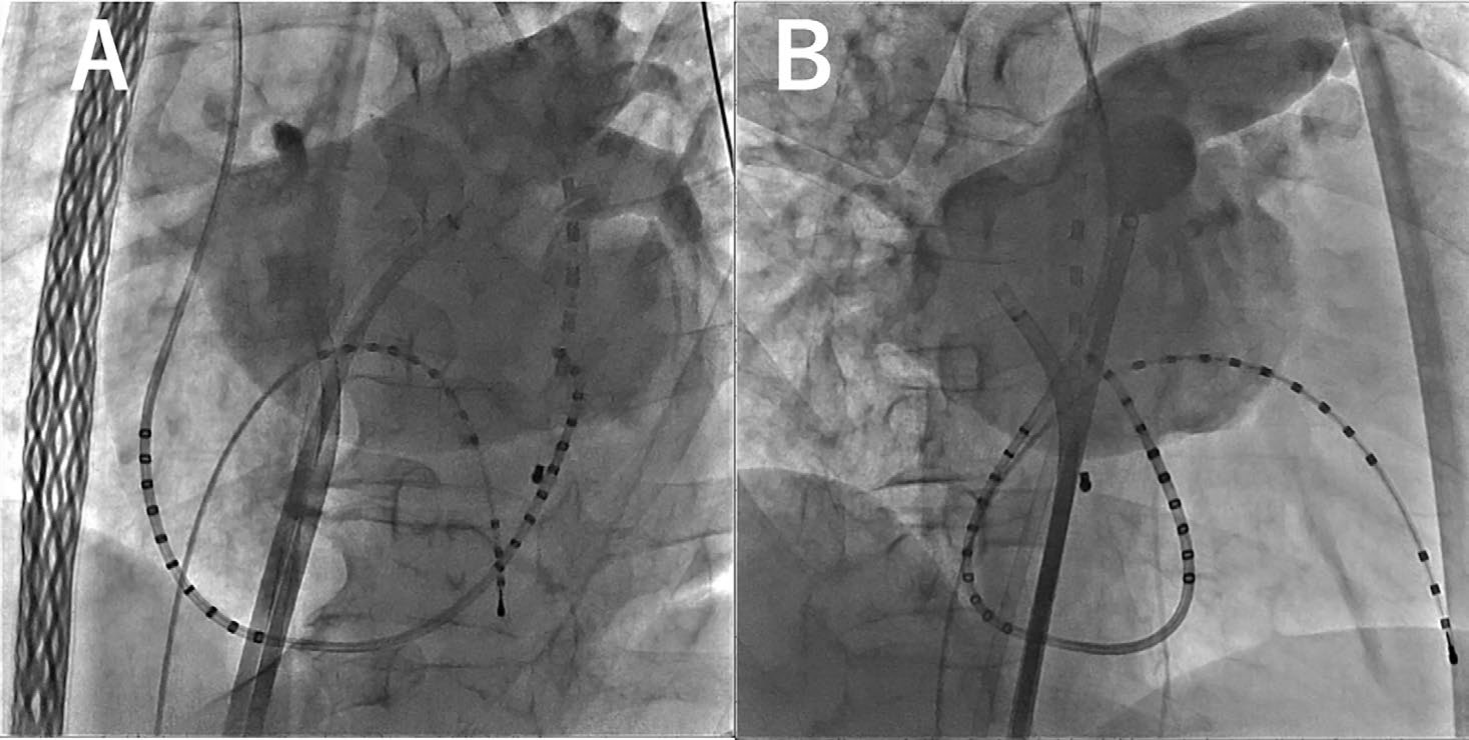
Left atrial angiography. Left (A) and right (B) anterior oblique views. Notably, the coronary sinus catheter is projected differently than usual.

Based on preoperative contrast‐enhanced CT findings, the radiofrequency (RF) needle (standard curve; Baylis Medical Technologies, Ontario, Canada) was precisely positioned at the level of the fossa ovalis and directed toward the vertebral body, specifically at the 6 o'clock position on the CT axial plane, which differs from the usual approach, under the guidance of intracardiac echocardiography (ICE) (Figure 2B, Video S1). The Brockenbrough procedure was performed with careful reference to preoperative contrast‐enhanced CT images to enhance the accuracy of the procedure and ensure the safety of the patient. An electroanatomic CARTO3 Mapping System and a CARTOSOUND Module (Biosense Webster, Diamond Bar, CA, USA) were used to reconstruct the geometry of the left atrium with circular catheters. Left atrial angiography was performed with burst pacing from the right ventricular catheter (Figure 3), and the geometry was merged with the anatomical data obtained via ICE.

**FIGURE 4:**
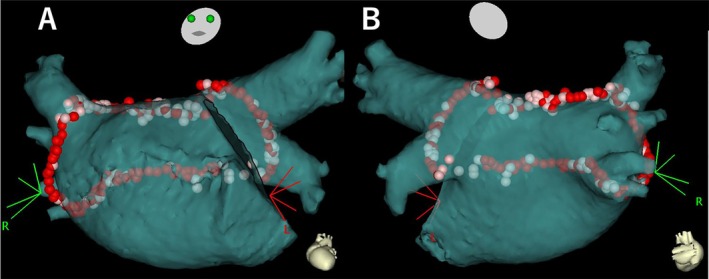
Box‐shaped pulmonary vein isolation. Box‐shaped pulmonary vein isolation to isolate all four pulmonary veins and the left atrial posterior wall as a single unit. The elimination of posterior wall potential was confirmed using a Lasso catheter. Anterior (A) and posterior (B) views. The red tags and pink tags represent the ablation lesions whose ablation indexes were more than 500 and 450–500, respectively.

Using a THERMOCOOL SMARTTOUCH Surround Flow (Biosense Webster) contact force‐sensing catheter, we delivered an RF energy of 30–40 W to the anterior and roof regions and 25–30 W to the posterior region of the PVs while measuring the contact force, which was maintained between 5 and 20 g for all RF applications with target ablation indices of 500 and 450, respectively. The LASSO (Biosense Webster) and Afocus catheters (St. Jude Medical, Little Canada, MN, USA) were positioned sequentially at the superior and inferior pulmonary veins, respectively. Subsequently, we used the double‐Lasso technique to confirm PV isolation [11] and applied RF energy to the anterior portion of each PV along with the left atrial roof and bottom regions to create a box‐shaped single ring encircling all PVs and LAPW (box‐PVI). After creating the circular lesion, we ascertained the disappearance of the electrograms from each PV and LAPW within the encircling line using a circular catheter (Figure 4). Thus, we confirmed the completion of entrance block of the box lesion and the absence of exit conduction upon high‐frequency stimulation (HFS), including dormant exit conduction using adenosine triphosphate, which can explain the robust lesion [12].

### 3.2 Cavotricuspid Isthmus Ablation

**FIGURE 5:**
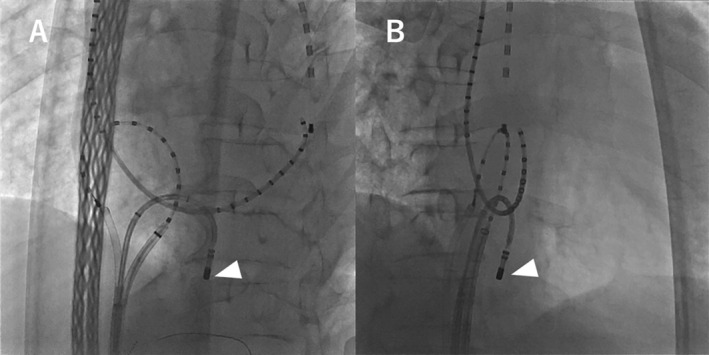
Cavotricuspid isthmus ablation. During cavotricuspid isthmus (CTI) ablation, the catheter position under fluoroscopy appeared significantly different from the usual due to anatomical variations. The white arrow indicates the ablation catheter. Left anterior oblique view (A) and right anterior oblique view (B).

We performed cavotricuspid isthmus (CTI) ablation following coronary sinus venography and right ventriculography owing to its unusual anatomical configuration. A pouch‐like structure was identified, which required a reverse catheter orientation and ablation approach (Figure 5). Despite these complexities, a bidirectional block across the CTI was successfully achieved, thus confirming the procedural endpoint.

## 4 Outcome and Follow‐Up

The patient was monitored postoperatively using continuous electrocardiographic surveillance. The electrocardiography (ECG) and echocardiography tests performed on the day after the procedure confirmed the maintenance of sinus rhythm, with no evidence of pericardial effusion suggestive of cardiac tamponade. The patient had an uneventful postoperative course and was discharged on postoperative day 3. For the first 3 months, the patient made outpatient visits once a month. Thereafter, regular medication prescriptions were delegated to a nearby clinic, and outpatient visits to our hospital were scheduled once a year. The patient's blood pressure remains well‐controlled without any issues. Every 3–6 months, 24‐h ambulatory ECG monitoring is performed in the outpatient setting. To date, over 8 years of follow‐up, the patient has had no AF recurrence.

## 5 Discussion

This report makes a novel contribution to the literature by underscoring the feasibility and potential utility of box‐PVI for patients with PE. Although similar considerations have been made in previous cases of PVI [8], anatomical variations associated with PE pose unique procedural challenges. In this case, considerable difficulties were encountered during the insertion of the coronary sinus (CS) catheter owing to the altered anatomical structure. CAG was performed to accurately identify the CS ostium and to guide catheter placement. Catheter insertion proved challenging owing to anatomical variations, necessitating careful reference to preoperative contrast‐enhanced CT images to ensure precise navigation and procedural success. Additionally, the atypical spatial relationship between the right and left atria makes the Brockenbrough procedure particularly challenging, even under ICE guidance, complicating transseptal puncture. Furthermore, sagittal compression between the sternum and vertebral bodies necessitated special attention to manipulating the catheters, even under contact force‐sensing monitoring, especially for delivering RF energy to the roof and bottom of the left atrium. These challenges were mitigated by the detailed anatomical imaging obtained via CT scan.

CTI linear ablation presented further difficulty owing to its atypical anatomical position and the presence of a pouch. Although CTI pouches can pose procedural difficulties in patients without PE, it has been hypothesized that the mechanical compression caused by PE potentially increases the likelihood of pouch formation [11]. Moreover, preoperative contrast‐enhanced CT revealed a significant atypical positional relationship between the inferior vena cava and tricuspid annulus, leading to probable procedural difficulty. Contrast injection from the CS was performed to accurately identify the CS ostium, and these results strongly correlated with preoperative contrast‐enhanced CT findings. Under normal circumstances, the ablation line is typically created at the 6–7 o'clock position in the left anterior oblique (LAO) view. However, in this case, owing to anatomical variations, the ablation line had to be created at the 4 o'clock position in the LAO view. Furthermore, the reverse hook approach was required to achieve optimal catheter positioning. Given the deviation from the standard ablation angle, the procedure was more technically challenging and required additional time to ensure precise lesion formation.

The box‐PVI technique offers a significant advantage over conventional PVI by incorporating linear ablation across the roof of the left atrium, particularly in cases with arrhythmogenic substrates other than the PV. In this case, conventional PVI was sufficiently feasible. However, because the posterior wall of the left atrium is compressed by the vertebral body in PE, this area could become arrhythmogenic. Therefore, we considered it desirable to isolate the posterior wall of the left atrium simultaneously. Supporting this notion, it has been reported that chronic mechanical compression from the sternum could induce myocardial fibrosis, potentially contributing to arrhythmogenicity at the right ventricular outflow tract in Brugada syndrome [13, 14]. A wider PVI including the LAPW, specifically box‐PVI, should be considered in such cases, even though it is more difficult to manipulate catheters when creating box‐PVI than when creating conventional ipsilateral PVI, as the compression and posterior displacement of the left atrial roof and floor reduce catheter stability and increase procedural difficulty. In the present case, the left atrial roof was notably longer and flatter than usual, posing additional challenges during ablation. Although the procedure was challenging, it was effective, as indicated by the absence of recurrence.

Despite the anatomical complexity, the procedure was successfully completed without complications based on a preoperative contrast‐enhanced CT to assess the intricate anatomy, which confirmed the contact force during the procedure, and utilizing 3D mapping for precise catheter navigation. In particular, during linear ablation of the left atrial roof, the catheter orientation and positional relationships differed from the usual approach, making contact force monitoring crucial. Given these anatomical challenges, meticulous intraoperative attention is imperative to minimize risks and ensure optimal outcomes.

## 6 Conclusions

We report a patient with AF and PE who underwent box‐PVI and experienced no recurrence of AF during an extended follow‐up period. This case underscores the feasibility and effectiveness of box‐PVI as a treatment for AF even in the presence of significant anatomical abnormalities associated with PE. The procedure demonstrated favorable long‐term outcomes, suggesting that box isolation may serve as a durable and reliable therapeutic option for managing AF in this unique subset of patients. For safe and effective box‐PVI ablation, obtaining detailed preoperative anatomical information using CT and various angiographic techniques, along with the integration of contact force‐sensing catheters and 3D imaging, is crucial.

## Author Contributions

**Takeshi Mori:** writing – original draft. **Kazuo Kato:** supervision, writing – review and editing. **Hiroko Goto:** writing – review and editing. **Shinjiro Miyata:** supervision.

## Consent

Written informed consent was obtained from the patient to publish this report in accordance with the journal's patient consent policy.

## Conflicts of Interest

The authors declare no conflicts of interest.

## Supporting information


**Video S1:** On contrast‐enhanced CT, the heart was highly compressed, and as shown in Figure 2B, the direction of Brockenbrough was toward the vertebral body, that is, at the 6 o'clock position.

## Data Availability

Data supporting the findings of this study are available from the corresponding author, Kazuo Kato, on reasonable request.
